# A preliminary world checklist of fern-mining insects

**DOI:** 10.3897/BDJ.9.e62839

**Published:** 2021-03-25

**Authors:** Jie Yang, Xuexiong Wang, Kevin Jan Duffy, Xiaohua Dai

**Affiliations:** 1 Leafminer Group, School of Life Sciences, Gannan Normal University, Ganzhou, China Leafminer Group, School of Life Sciences, Gannan Normal University Ganzhou China; 2 Institute of Systems Science, Durban University of Technology, Durban, South Africa Institute of Systems Science, Durban University of Technology Durban South Africa; 3 National Navel-Orange Engineering Research Center, Ganzhou, China National Navel-Orange Engineering Research Center Ganzhou China

**Keywords:** fern, leaf miner, host plant, plant-insect interactions, plant apparency hypothesis

## Abstract

Compared to the leaf-miners and stem-miners on flowering plants, the miners on ferns (including both Lycopodiophyta and Polypodiophyta in the broad sense) are less known. Knowledge of miners and their host plants is essential to fully understand plant-insect interactions. Although there are many scattered records on fern miners, a worldwide checklist has not been reported.

We provide a preliminary checklist of fern-mining insects and their host plants worldwide. Altogether, we found records for 128 species and 18 families of fern miners, mainly that feed on Dennstaedtiaceae, Equisetaceae, Polypodiaceae and Aspleniaceae. Fern miners belonged to four orders: Diptera (51 species; 39.8%), Coleoptera (33 species; 25.8%), Lepidoptera (28 species; 21.9%) and Hymenoptera (16 species; 12.5%). They are primarily known from the Palaearctic Region, Nearctic Region, Neotropical Region and Oriental Region.

## Introduction

Leaf/stem miners are endophagous insects whose larvae feed on parenchyma or epidermal cells and form visually distinctive feeding tunnels, i.e. ‘mines’ on the leaves or stems (Dai et al. 2018, Eiseman 2020b, Liu et al. 2015, Sinclair and Hughes 2010). The mines can provide useful hints on insect species identities, insect life histories, insect behaviour and insect-plant interactions (Dai et al. 2018). Fewer miner groups (e.g. gracillariid moths, agromyzid flies and leaf-mining chrysomelids) can utilise over 100 plant families (Dai et al. 2017, De Prins and De Prins 2020, Santiago-Blay 2004, Spencer 1990), which are mainly angiosperm families, such as Fagaceae and Myrtaceae (Dai et al. 2018).

Ferns (Pteridophyta, including both Lycopodiophyta and Polypodiophyta in the broad sense) are the second largest group of vascular plants, just after angiosperms (Dai et al. 2020, Schneider et al. 2004). With lower nutrition, higher defensive chemicals and no flowers, the interspecific associations between ferns and insects are often overlooked (Mehltreter et al. 2010, Weintraub et al. 1995). However, ferns used to be the primary food resource for insects before the thriving of angiosperms (Mehltreter et al. 2010). In fact, fossil records indicate that ferns and insects have co-evolved for at least 300 myr (Chandra and Srivastava 2003). Some ferns have nectaries and domatia, which could attract ants to be bodyguards (Mehltreter et al. 2010). Moreover, some insects mimic the soral crypsis of ferns to escape from their natural enemies (Barker et al. 2005, Patra et al. 2008). Some researchers hypothesise that fern-feeding insects should have fewer species, genera and families than those of seed plants (Weintraub et al. 1995), while others suggest that the richness of fern-feeding insects is largely underestimated (Auerbach and Hendrix 1980, Mehltreter et al. 2010). The possible underestimation might be deduced from the following facts: (1) many fewer investigations have been performed for wild ferns than for cultivated ferns or invasive ferns (Fountain-Jones et al. 2012); (2) many more fern herbivores have been discovered in the comprehensive screening of bio-control agents for pest ferns (Mehltreter et al. 2010); (3) no noticeable difference has been found between leaf herbivory loss of ferns and that of seed plants (Chandra and Srivastava 2003); and (4) the possible biases of plant apparency (i.e. body size, distribution range and individual numbers (Dai et al. 2017)) are not considered for phytophagous insects on ferns in comparison to those on seed plants (Auerbach and Hendrix 1980).

Fern-feeding insects could be classified as generalists and specialists. Most fern-feeding generalists tend to be classified as leaf-chewing or sap-sucking, while most specialists are classified as leaf-mining, gall-forming or spore-feeding (Mehltreter et al. 2010). By far, the miners on ferns are much less known than those on seed plants; although there are scattered records on publications and websites (De Prins and De Prins 2020, Edmunds et al. 2020, Ellis 2020, Eiseman 2020b, Pitkin et al. 2019, Santiago-Blay 2004, Spencer 1990), few comprehensive reviews on fern miners have been provided and a worldwide checklist has not been reported. In this study, we will compile a preliminary checklist of fern-mining insects and their host plants throughout the world, which could provide meaningful information to the study of plant-insect–environment interactions.

## Material and methods

The names and hosts of fern miners were obtained from websites, books and articles. Most publications were retrieved from the Web of Science (https://www.webofknowledge.com) and Google Scholar (https://scholar.google.com), while the others were obtained from reference lists of the websites and retrieved publications. According to the Taxonomic Name Resolution Service (http://tnrs.iplantcollaborative.org/TNRSapp.html), the host fern's scientific names were verified and corrected. The number of species in a fern family was obtained from the Catalogue of Life (http://www.catalogueoflife.org/). Based on two recent mega-trees (Smith and Brown 2018, Zanne et al. 2014), 'GBOTB.extended.tre' is the latest and largest dated phylogenetic tree for vascular plants, with 74533 species, 10587 genera and all extant 479 families (Jin and Qian 2019). The R package 'V. PhyloMaker' (Jin and Qian 2019) can bind undetermined plant taxa to the backbone phylogeny of 'GBOTB.extended.tre' and generates the customised tree we needed (Dai et al. 2020). Here, we obtained the phylogenetic tree of our host fern families using the above method. Bivariate linear regression was fitted with Past 4.04 (Hammer et al. 2001).

Both leaf-miners and stem-borers have been found in the same insect family (e.g. Buprestidae, Cossidae and Blasticotomidae) or even in the same genus (e.g. *Amauromyza*, *Melanagromyza*, *Phytomyza* and *Zygoneura*) (Eiseman 2020b, Hering 1951, Shcherbakov 2006, Woodley and Janzen 1995). Occasionally, the same species could change their feeding habits from leaf-mining to stem-mining or stem-boring, when the younger larvae transform into the older larvae, when the leaf is too small to offer enough food or when leaves and stems do not differ significantly (Eiseman 2020b, Hering 1951). Such phenomena can be found in *Heliozela hammoniella* (= *Heliozela betulae*) (Heliozelidae), *Marmara* spp. (Gracillariidae), *Ophiomyia* spp. (Agromyzidae), *Phyllocnistis* spp. (Gracillariidae), *Scaptomyza graminum* (= *Scaptomyzella incana*) (Drosophilidae), *Zygoneura calthella* (Sciaridae), *Temnosira czurhini* (Pallopteridae) and many other species (Eiseman 2020b, Ellis 2020, Hering 1951, Kato 2002). There are transitions amongst leaf-mining, stem-mining, leaf-boring and stem boring (Hering 1951). Moreover, most ferns are herbaceous, with developed parenchyma in the stems (Crang et al. 2018). Therefore, we incorporated fern borers into fern miners for this article (Suppl. material 1). Some suspected insect species are not included in this study (e.g. Correia et al. 2020, Santiago-Blay 2004).

The miners' biogeographical regions followed Juan J. Morrone's system (Morrone 2002). For detailed information about fern miners associated with each host plant species, the original sources should be consulted.

## Results

We recorded 128 species and 18 families of fern miners (Table 1; Suppl. material 2), including Agromyzidae, Anthomyiidae, Drosophilidae, Chironomidae, Pallopteridae, Buprestidae, Chrysomelidae, Curculionidae, Crambidae, Noctuidae, Tineidae, Tortricidae, Cosmopterigidae, Gelechiidae, Hepialidae, Psychidae, Blasticotomidae and Tenthredinidae. They were primarily distributed in the Palaearctic Region, Nearctic Region and Oriental Region of the Northern Hemisphere and the Neotropical Region between the Tropic of Cancer and the Tropic of Capricorn (Table 1). One explanation for this distribution pattern could be that the land area in the Northern Hemisphere is almost double that of the Southern Hemisphere. Another reason might be that the investigations on leaf-mining insects and their host plants are more thorough in the Northern than in the Southern Hemisphere (Sinclair and Hughes 2008, Sinclair and Hughes 2008, Sinclair and Hughes 2010).

**Figure 1.**
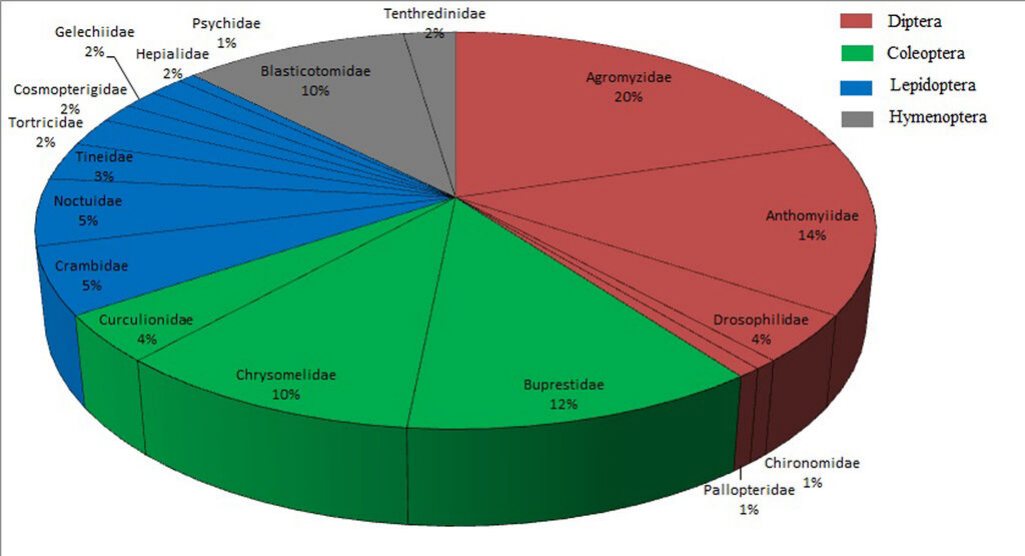
Percentage distribution of the fern-mining species into the four orders and the 18 families.

Fern miners belong to four orders: Diptera (51 species; 39.8%), Coleoptera (33 species; 25.8%), Lepidoptera (28 species; 21.9%) and Hymenoptera (16 species; 12.5%) (Fig. 1; Suppl. material 2). In general, dipteran leaf miners are dominant in herbaceous plants while lepidopteran leaf miners are dominant in woody plants (De Prins and De Prins 2020, Edmunds et al. 2020, Eiseman 2020b, Ellis 2020, Pitkin et al. 2019, Spencer 1990). The life form of most extant ferns is herbaceous, which could explain why nearly half of fern-mining species are dipteran flies.

Amongst the 18 fern-mining insect families, Agromyzidae, Anthomyiidae, Buprestidae, Chrysomelidae and Blasticotomidae had the highest numbers of species (20.3%, 14.1%, 11.7%, 10.2% and 10.2%, respectively), while the other 13 families accounted for 33.5% only (Fig. 1; Suppl. material 2).

**Figure 2.**
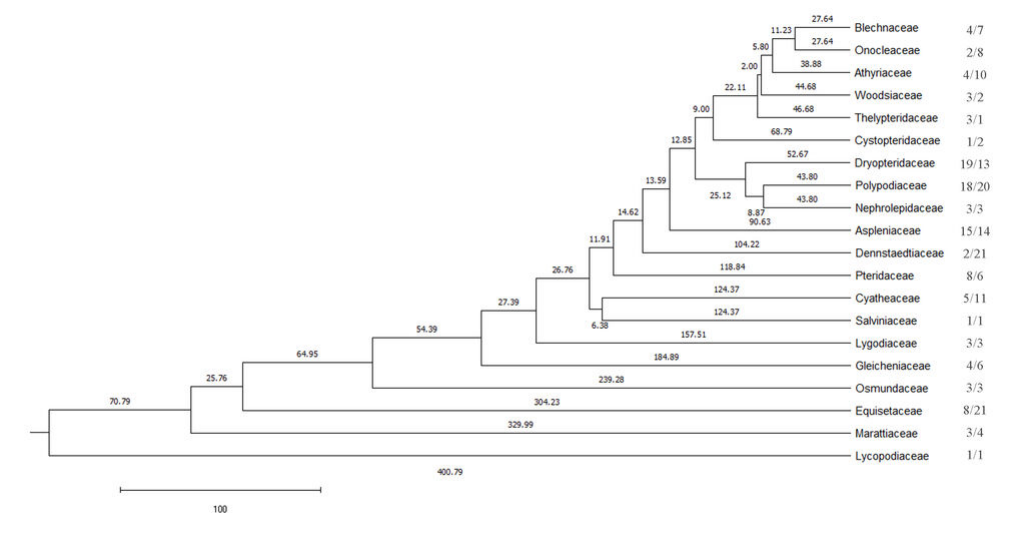
The dated phylogenetic tree of host fern families generated with the R package 'V. PhyloMaker' (Jin and Qian 2019). The first number after the fern family is the number of host fern species and the second is the number of fern miner species. The length of each branch is also shown and the scale bar unit is 100 myr.

The fern families with highest numbers of host species were Dryopteridaceae (19), Polypodiaceae (18) and Aspleniaceae (15) (Fig. 2; Suppl. material 3). The fern families with the highest numbers of miner species were Dennstaedtiaceae (21), Equisetaceae (21), Polypodiaceae (20) and Aspleniaceae (14) (Fig. 2; Suppl. material 3). With 82 species and 12 families of host ferns and 67 species of fern miners, Polypodiales was the dominant host order of fern-mining insects (Suppl. material 3).

The number of host species was significantly and positively correlated with the total number of fern species at the family level (*R*^2^ = 0.614, *t* = 5.352, *P* < 0.001; Fig. 3a), but the number of miner species was not significantly correlated with the total number of fern species at the family level (*R*^2^ = 0.110, *t* = 1.495, *P* = 0.152; Fig. 3b).

## Discussion

In this paper, we provide a preliminary checklist about fern miners and their host plants worldwide. Table 1 summarises this checklist in terms of published information to date. However, there is also more information available on some fern-mining groups and this is summarised here:

(1) Diptera: In Anthomyiidae, there is an unknown *Chirosia* species with *Deparia acrostichoides* as host in the Nearctic Region (Eiseman 2020b), while *C. similata* could be a possible Nearctic *Pteridium* borer (Eiseman 2018). In Drosophilidae, the Fuscoamoeba subgroup has many species that have been reared from rotting fern rachises (Magnacca et al. 2008). For *Chromatomyia* species in Agromyzidae, Kahanpää (2014) separates *Chromatomyia* and *Napomyza* as different genera (Kahanpää 2014) and Spencer (1990) considers that *C. cheilanthus* should belong to the genus *Ptochomyza* (Spencer 1990). Molecular phylogeny suggests that the genus of *Phytomyza* should include all species of *Phytomyza*, *Chromatomyia*, *Napomyza* and *Ptochomyza* (Winkler et al. 2009). However, only one fern-feeding *Chromatomyia* species is included in the above molecular analysis. Moreover, no *Phytomyza* *s. s.* species has previously been found as fern-mining. In this article, we rather kept the genus name of *Chromatomyia* and listed the *Phytomyza* species as the synonym of the corresponding *Chromatomyia* species in Table 1.

(2) Lepidoptera: In Tineidae, early instar larvae of the subfamily Teichobinae are leaf miners, while their later instars feed on sporangia from a loose portable case (Gaedike 2019). An unknown species of Pyralidae has two hosts (*Lygodium microphyllum* and *L. flexuosum*) in the Oriental Region (Goolsby et al. 2003). There is an unknown moth in the Nearctic Region, which mines the leaves of *Pteridium aquilinum* (Eiseman 2020b), but the species name could not be confirmed. In Gelechiidae, *Monochroa placidella* larvae make gall-like deformities on the fronds of the bracken (*P. aquilinum*) (Eiseman 2020b). Eiseman (personal observations) believes that the deformities are caused by internal feeding; he has also reared an undetermined *Monochroa* species from larvae that similarly bored in the terminal part of the rachis and caused a gall-like deformity.

(3) Hymenoptera: In Tenthredinidae, the genus *Heptamelus* has 36 species in the Palaearctic and Oriental Regions and their larvae are internal feeders and all probably use ferns as larva hosts (Vikberg and Liston 2009), but we cannot know with certainty which species of *Heptamelus* is involved, except for *H. ochroleucus* on *Athyrium filix-femina* (Vikberg 2017). With only 13 species and 3 tribes in Eurasia's temperate region, Blasticotomidae is a small family in the Hymenoptera and their larvae are stem borers on ferns (Taeger et al. 2010, Wikipedia 2019, Santiago-Blay 2004).

(4) Coleoptera: In Buprestidae, both *Neotrachys* and *Endelus* have fern-mining habits (Xiao 2018, Bellamy 1997). Most *Neotrachys* feed on the ferns of Cyatheaceae and Gleicheniaceae (Hespenheide 1980, Hespenheide 1982, Hespenheide 2006). However, some *Neotrachys* larvae may mine other non-fern plants. For example, *N. dominicanus* feeds on *Arthrostylidium* (Poaceae) (Meurgey 2017). The genus *Leiopleura* is morphologically similar and sometimes confused with *Neotrachys*, but *Leiopleura* feeds on Moraceae and Apocynaceae (Hespenheide 1991). Fern-feeding or not could be a clue to distinguish *Neotrachys* and its related genera. Although there are many publications on *Endelus*, only very few mention its host plants (Kalashian 2013).

Dominant plant groups generally are rich in leaf miners and rich in host plants, which could be explained by the ‘plant apparency hypothesis’ (Feeny 1976). Such phenomena have been found in several other leaf-mining insects (Dai et al. 2017, Dai et al. 2018). Apart from species richness in a fern taxonomic group, the distribution range should also be considered as an important component of 'plant apparency' (Dai et al. 2017). Equisetaceae has 39 species and eight host species, and Dennstaedtiaceae has 245 species and two host species, but both families host 21 miner species, which is the highest amongst all fern families (Fig. 2; Suppl. material 3). It is Equisetaceae and Dennstaedtiaceae that strongly affected the significance of the correlation in Fig. 3b. In particular, the bracken fern (*Pteridium aquilinum*), one species in Dennstaetiaceae, had 20 miner species (Suppl. material 3), which is not less than many dominant flowering plants. The bracken fern might be the only globally distributed fern and one of the most widespread vascular plants, which occurs in temperate and subtropical regions in both hemispheres (Flora of North America Editorial Committee 1993). It is used as vegetable, food or feed in many places. It is also a common invasive plant in disturbed areas (Flora of North America Editorial Committee 1993). The above features of the bracken fern make it highly attractive to both miners and researchers, thus the high number of mining species might be the combined effects of plant apparency and sampling effects. Dryopteridaceae has 2257 species (Suppl. material 3) and also a cosmopolitan distribution, with many cultivated ornamental species (Olsen and Olsen 2007). Aspleniaceae has 855 species (Suppl. material 3) and also a worldwide distribution (POWO 2019). Polypodiaceae has 1667 species (Suppl. material 3) and is distributed nearly worldwide, but mainly in tropical areas, with some cultivated species (Simpson 2010). Both high species richness and wide geographical distribution could explain why the three families have large numbers of both host fern species and miner species. Besides *P. aquilinum*, *Equisetum arvense* (Equisetaceae), *Athyrium filix-femina* (Athyriaceae) and *Matteuccia struthiopteris* (Onocleaceae) also have a high richness of miners (10, 8 and 6 species, respectively) (Suppl. material 3). The common horsetail (*E. arvense*) is native throughout the Arctic and temperate regions in the Northern Hemisphere (Schaffner 1930). *E. arvense* becomes an invasive plant in New Zealand and a systematic evaluation of its potential biocontrol agents including miners and borers has been performed (Paynter and Barton 2008). The common lady-fern (*A. filix-femina*) is one of the most abundant fern species in the temperate regions in the Northern Hemisphere (Adam 1995). The ostrich fern (*M. struthiopteris*) is widely distributed in the temperate regions of the Northern Hemisphere (Kimura et al. 2004). However, since the checklist of fern-mining insects and the corresponding host fern species is preliminary, these patterns need further verification.

As the sampling of fern miners and their hosts are insufficient in many places and some sampled records might be inaccessible, this study was only a preliminary list. We hope that this basic list can serve as an inital reference for future inventories and research on fern-mining insects.

## Supplementary Material

3CE796E3-827F-5CC4-9BCA-493C03FD60D0 10.3897/BDJ.9.e62839.suppl1 Supplementary material 1 The feeding mode and feeding plant organ of each fern miner Data type Feeding habits File: oo_522267.xlsx https://binary.pensoft.net/file/522267 Jie Yang, Xiaohua Dai

89FA8EE3-A356-53F5-BD9E-09999AC27F32 10.3897/BDJ.9.e62839.suppl2 Supplementary material 2 The number of fern-miners in each insect family and each insect order Data type Number of species File: oo_522268.xlsx https://binary.pensoft.net/file/522268 Jie Yang, Xiaohua Dai

2446628C-E69F-54DB-8863-91B14BAAA21C 10.3897/BDJ.9.e62839.suppl3 Supplementary material 3 The number of miners on each fern species, each fern family and each fern order Data type Number of species File: oo_522269.xlsx https://binary.pensoft.net/file/522269 Jie Yang, Xiaohua Dai

## Figures and Tables

**Figure 3a.**
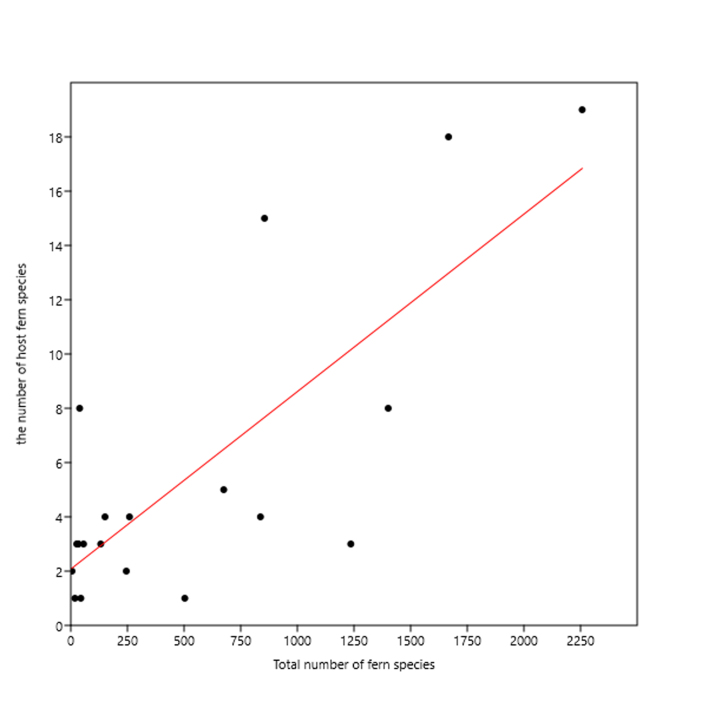


**Figure 3b.**
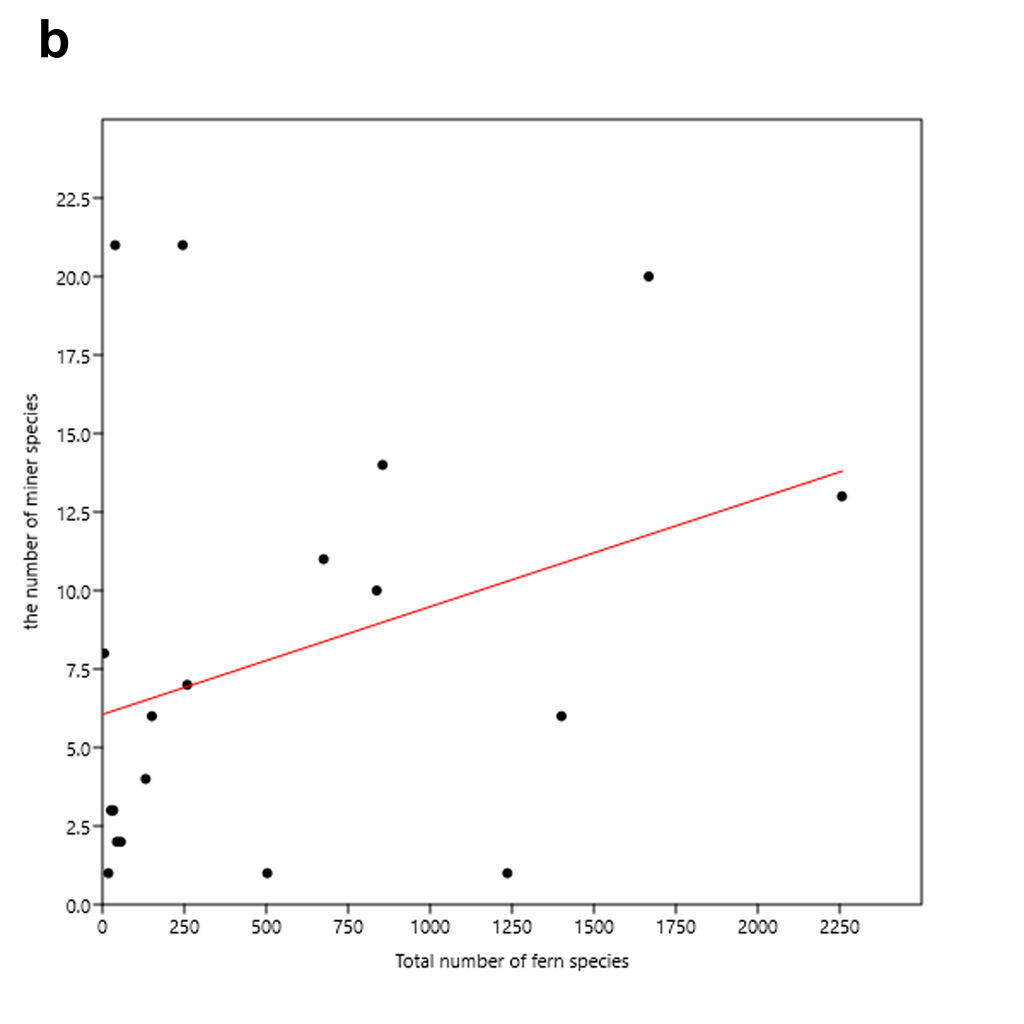


**Table 1.** A preliminary world checklist of fern miners and their biological information.

**Miner family**	**Miner species**	**Host fern**	**Biogeographical regions**	**References**
** Diptera **				
Agromyzidae	*Chromatomyia cheilanthus* Garg*^1^	*Cheilanthes virga-aure*	Oriental Region	Spencer 1990
Agromyzidae	*Chromatomyia dorsata* Hendel^2^	*Asplenium ceterach*	Palaearctic Region	Ellis 2020, Spencer 1990
Agromyzidae	*Chromatomyia dryoptericola* Sasakawa^3^	*Dryopteris lacera*	Palaearctic Region	Sasakawa 2010, Spencer 1990
Agromyzidae	*Chromatomyia dryoptericola* Sasakawa	*Asplenium pinnatifidum*	Palaearctic Region	
Agromyzidae	*Chromatomyia dryoptericola* Sasakawa	*Lepisorus thunbergianus*	Palaearctic Region	
Agromyzidae	*Chromatomyia masumiae* Sasakawa	*Lepisorus thunbergianus*	Palaearctic Region	Sasakawa 2010
Agromyzidae	*Chromatomyia scolopendri* Robineau Desvoidy^4^	*Asplenium ruta-muraria*	Palaearctic Region	Civelek 2002, Dempewolf 2001, Ellis 2020, Sasakawa 2010, Spencer 1990
Agromyzidae	*Chromatomyia scolopendri* Robineau Desvoidy	*Asplenium scolopendrium*	Palaearctic Region	
Agromyzidae	*Chromatomyia scolopendri* Robineau Desvoidy	*Asplenium septentrionale*	Palaearctic Region	
Agromyzidae	*Chromatomyia scolopendri* Robineau Desvoidy	*Polypodium vulgare*	Palaearctic Region	
Agromyzidae	*Liriomyza equiseti* de Meijere^5^	*Equisetum arvense*	Nearctic and Palaearctic Regions	Eiseman 2020b, Ellis 2020, George 2014, Lonsdale 2017, Spencer 1990
Agromyzidae	*Liriomyza occipitalis* Hendel^6^	*Equisetum arvense*	Palaearctic Region	Ellis 2020, George 2014, Spencer 1990
Agromyzidae	*Liriomyza nordica* Spencer*	*Equisetum* sp.	Nearctic Region	Eiseman 2020b
Agromyzidae	*Liriomyza virgo* Zetterstedt^7^	*Equisetum fluviatile*	Nearctic and Palaearctic Regions	Eiseman 2020b, Ellis 2020, George 2014, Lonsdale 2017, Spencer 1990
Agromyzidae	*Liriomyza virgo* Zetterstedt	*Equisetum palustre*	Nearctic and Palaearctic Regions	
Agromyzidae	*Liriomyza virgula* Frey^8^	*Equisetum arvense*	Palearctic Regain	George 2014
Agromyzidae	*Phytoliriomyza clara* Melander	*Pteridium aquilinum*	Nearctic Region	Eiseman 2020b, Spencer 1990
Agromyzidae	*Phytoliriomyza cyatheae* Spencer	*Cyathea dealbata*	Neozelandic Region	Spencer 1976, Spencer 1990
Agromyzidae	*Phytoliriomyza cyatheae* Spencer	*Cyathea smithii*	Neozelandic Region	
Agromyzidae	*Phytoliriomyza diplazii* Sasakawa	* Diplazium *	Oriental Region	Spencer 1990
Agromyzidae	*Phytoliriomyza felti* Malloch	*Asplenium pinnatifidum*	Nearctic Region	Eiseman 2020b, Spencer 1990
Agromyzidae	*Phytoliriomyza felti* Malloch	*Asplenium platyneuron*	Nearctic Region	
Agromyzidae	*Phytoliriomyza felti* Malloch	*Pellaea atropurpurea*	Nearctic Region	
Agromyzidae	*Phytoliriomyza felti* Malloch	*Pellaea glabella*	Nearctic Region	
Agromyzidae	*Phytoliriomyza felti* Malloch	*Asplenium rhizophyllum*	Nearctic Region	
Agromyzidae	*Phytoliriomyza felti* Malloch	*Woodsia obtusa*	Nearctic Region	
Agromyzidae	*Phytoliriomyza flavopleura* Watt	* Microsorum *	Neozelandic Region	Spencer 1990
Agromyzidae	*Phytoliriomyza flavopleura* Watt	*Notogrammitis billardierei*	Neozelandic Region	
Agromyzidae	*Phytoliriomyza flavopleura* Watt	*Asplenium flaccidum*	Neozelandic Region	
Agromyzidae	*Phytoliriomyza flavopleura* Watt	*Asplenium oblongifolium*	Neozelandic Region	
Agromyzidae	*Phytoliriomyza hilarella* Zetterstedt	* Asplenium *	Palaearctic Region	Ellis 2020, Lawton 1982, Lawton 1976, MacGarvin et al. 1986, Rigby and Lawton 1981, Spencer 1990
Agromyzidae	*Phytoliriomyza hilarella* Zetterstedt	*Pteridium aquilinum*	Palaearctic Region	
Agromyzidae	*Phytoliriomyza hilarella* Zetterstedt	* Polypodium *	Palaearctic Region	
Agromyzidae	*Phytoliriomyza kuscheli* Spencer	* Histiopteris *	Oriental Region	Spencer 1990
Agromyzidae	*Phytoliriomyza pteridii* Spencer	*Pteridium aquilinum*	Palaearctic Region	Gerson 1979, Mcgavin and Brown 1986, MacGarvin et al. 1986, Spencer 1990
Agromyzidae	*Phytoliriomyza pulchella* Spencer*	*Pteridium aquilinum*	Nearctic Region	Eiseman 2020b
Agromyzidae	*Phytoliriomyza* sp1	Marattia*	Oriental Region	Spencer 1990
Agromyzidae	*Phytoliriomyza* sp2	* Cyathea *	Neotropical and Andean Regions	Spencer 1990
Agromyzidae	*Phytoliriomyza tearohensis* Spencer	*Cyathea dealbata*	unknown	Spencer 1976, Spencer 1990
Agromyzidae	*Tropicomyia angioptericola* Shiao	*Angiopteris lygodiifolia*	Palaearctic Region	Shiao and Wu 2005
Agromyzidae	*Tropicomyia polyphaga* Spencer	* Nephrolepis *	Oriental Region	Spencer 1990, Shiao and Wu 2005
Agromyzidae	*Tropicomyia* sp1	* Pleopeltis *	Afrotopical Region	Spencer 1990
Agromyzidae	*Tropicomyia* sp1	*Asplenium auriculatum*	Afrotopical Region	
Agromyzidae	*Tropicomyia* sp2	Angiopteris evecta*	Oriental Region	Spencer 1990
Anthomyiidae	*Chirosia albifrons* Tiens	*Pteridium aquilinum*	Palaearctic Region	Lawton 1976, MacGarvin et al. 1986
Anthomyiidae	*Chirosia albitarsis* Zetterstedt	*Pteridium aquilinum*	Palaearctic and Oriental Regions	Ellis 2020, Gerson 1979, Lawton 1976, Mcgavin and Brown 1986, Suwa 1984, Suwa 1999
Anthomyiidae	*Chirosia asperistilata* Suwa	*Dryopteris monticola*	Palaearctic Region	Suwa 1999, Suwa 2005
Anthomyiidae	*Chirosia asperistilata* Suwa	*Dryopteris crassirhizoma*	Palaearctic Region	
Anthomyiidae	*Chirosia cinerosa* Zetterstedt^9^	*Pteridium aquilinum*	Palaearctic Region	Ellis 2020, Kwon and Suh 1982, Suwa 1999
Anthomyiidae	*Chirosia cinerosa* Zetterstedt	*Matteuccia struthiopteris*	Palaearctic Region	
Anthomyiidae	*Chirosia cinerosa* Zetterstedt	*Athyrium filix-femina*	Palaearctic Region	
Anthomyiidae	*Chirosia crassiseta* Stein	*Pteridium aquilinum*	Palaearctic Region	Brown and McGavin 2007, Ellis 2020, Gerson 1979, Lawton 1976, Mcgavin and Brown 1986
Anthomyiidae	*Chirosia filicis* Huckett	*Osmunda claytoniana*	Nearctic Region	Eiseman 2018, Eiseman 2020b
Anthomyiidae	*Chirosia filicis* Huckett	*Osmundastrum cinnamomeum*	Nearctic Region	
Anthomyiidae	*Chirosia flavipennis* Fallen	*Pteridium aquilinum*	Nearctic and Palaearctic Regions	Eiseman 2020b, Eiseman 2018, Lawton 1976, Suwa 2013
Anthomyiidae	*Chirosia gleniensis* Huckett	*Onoclea sensibilis*	Nearctic Region	Eiseman 2020b, Eiseman 2018, Eiseman 2020a
Anthomyiidae	*Chirosia gleniensis* Huckett	*Woodsia areolata*	Nearctic Region	
Anthomyiidae	*Chirosia gleniensis* Huckett	Woodsia virginica*	Nearctic Region	
Anthomyiidae	*Chirosia griseifrons* Séguy	Dryopteris*	Palaearctic Region	Ellis 2020, Suwa 1999
Anthomyiidae	*Chirosia griseifrons* Séguy	*Athyrium filix-femina*	Palaearctic Region	
Anthomyiidae	*Chirosia grossicauda* Strobl^10^	* Asplenium *	Palaearctic Region	Ellis 2020, Gerson 1979, Lawton 1976, MacGarvin et al. 1986, Mcgavin and Brown 1986, Suwa 1999
Anthomyiidae	*Chirosia grossicauda* Strobl	*Pteridium aquilinum*	Palaearctic Region	
Anthomyiidae	*Chirosia grossicauda* Strobl	Dryopteris*	Palaearctic Region	
Anthomyiidae	*Chirosia histricina* Rondani^11^	*Osmunda regalis*	Nearctic and Palaearctic Regions	Brown and McGavin 2007, Ellis 2020, MacGarvin et al. 1986, Mcgavin and Brown 1986, Suwa 1999
Anthomyiidae	*Chirosia histricina* Rondani	* Asplenium *	Nearctic and Palaearctic Regions	
Anthomyiidae	*Chirosia histricina* Rondani	*Blechnum spicant*	Nearctic and Palaearctic Regions	
Anthomyiidae	*Chirosia histricina* Rondani	*Pteridium aquilinum*	Nearctic and Palaearctic Regions	
Anthomyiidae	*Chirosia histricina* Rondani	*Dryopteris filix-mas*	Nearctic and Palaearctic Regions	
Anthomyiidae	*Chirosia histricina* Rondani	*Matteuccia struthiopteris*	Nearctic and Palaearctic Regions	
Anthomyiidae	*Chirosia histricina* Rondani	* Polypodium *	Nearctic and Palaearctic Regions	
Anthomyiidae	*Chirosia histricina* Rondani	*Athyrium filix-femina*	Nearctic and Palaearctic Regions	
Anthomyiidae	*Chirosia histricina* Rondani	*Cystopteris fragilis*	Nearctic and Palaearctic Regions	
Anthomyiidae	*Chirosia montana* Pokorny	*Cystopteris fragilis*	Nearctic and Palaearctic Regions	Eiseman 2020b, Eiseman 2018
Anthomyiidae	*Chirosia nigripes* Bezzi	*Pteridium aquilinum*	Palaearctic Region	Ellis 2020, Suwa 1999
Anthomyiidae	*Chirosia pusillans* Huckett	*Athyrium filix-femina*	Nearctic Region	Eiseman 2018, Eiseman 2020a, Eiseman 2020b
Anthomyiidae	*Chirosia pusillans* Huckett	*Matteuccia struthiopteris*	Nearctic Region	
Anthomyiidae	*Chirosia spinosissima* Malloch	*Pteridium aquilinum*	Nearctic and Palaearctic Regions	Eiseman 2020b, Eiseman 2020a
Anthomyiidae	*Pegomya cedrica* Huckett	*Equisetum hyemale*	Nearctic Region	Michelsen and Palmer 2020
Anthomyiidae	*Pegomya disticha* Griffiths	*Equisetum hyemale*	Nearctic Region	Michelsen and Palmer 2020
Anthomyiidae	*Pegomya glabra* Stein	* Equisetum *	Nearctic Region	Michelsen and Palmer 2020
Drosophilidae	*Drosophila apicipuncta* Hardy	* Sadleria *	Nearctic Region	Magnacca et al. 2008, Magnacca and O'Grady 2014, Maunsell et al. 2016
Drosophilidae	*Drosophila diminuens* Hardy*	* Sadleria *	Nearctic Region	Magnacca and O'Grady 2014
Drosophilidae	*Drosophila sadleria* Bryan	* Sadleria *	Nearctic Region	Magnacca et al. 2008
Drosophilidae	*Scaptodrosophila notha* Bock	*Pteridium aquilinum*	Australotropical and Australotemperate Regions	Maunsell et al. 2016
Drosophilidae	*Scaptodrosophila* sp.	*Parablechnum wattsii*	Australotropical and Australotemperate Regions	Maunsell et al. 2016
Chironomidae	*Bryophaenocladius furcatus* Kieffer	* Adiantum *	Nearctic and Palaearctic Regions	Eiseman 2020b
Pallopteridae	*Temnosira czurhini* Ozerov	*Huperzia serrata*	Palaearctic Region	Kato 2002
** Lepidoptera **				
Crambidae	*Albusambia elaphoglossumae* Solis & Davis	*Elaphoglossum conspersum*	Nearctic Region	Solis et al. 2005a
Crambidae	*Albusambia elaphoglossumae* Solis & Davis	*Elaphoglossum biolleyi*	Nearctic Region	
Crambidae	*Eudonia zophoclaena* Meyrick	*Pyrrosia eleagnifolia*	Neozelandic Region	Patrick 2015
Crambidae	*Scoparia illota* Philpott	*Pyrrosia eleagnifolia*	Neozelandic Region	Patrick 2015
Crambidae	*Scoparia molifera* Meyrick	*Pyrrosia eleagnifolia*	Neozelandic Region	Patrick 2015
Crambidae	*Siamusotima aranea* Solis & Yen	*Lygodium flexuosum*	Oriental Region	Solis et al. 2005b
Crambidae	*Siamusotima disrupta* Solis	* Lygodium *	Palaearctic Region	Solis et al. 2017
Crambidae	*Undulambia polystichalis* Capps	*Rumohra adiantiformis*	Nearctic Region	Gerson 1979
Noctuidae	*Hydraecia micacea* Esper	* Equisetum *	Palearctic Region	Ellis 2020
Noctuidae	*Papaipema inquaesita* Grote & Robinson	*Onoclea sensibilis*	Nearctic Region	Bird 2012
Noctuidae	*Papaipema pterisii* Bird	*Pteridium aquilinum*	Nearctic Region	Bird 2012, [Bibr B6425320][Bibr B6773896]
Noctuidae	*Papaipema pterisii* Bird	Matteuccia struthiopteris*	Nearctic Region	
Noctuidae	*Papaipema speciosissima* Grote & Robinson	*Osmunda regalis*	Nearctic Region	Hinz and Zahniser 2015, Lafontaine and Schmidt 2010, Oppenheim et al. 2018
Noctuidae	*Papaipema speciosissima* Grote & Robinson	*Osmundastrum cinnamomeum*	Nearctic Region	
Noctuidae	*Papaipema stenocelis* Dyar	*Woodwardia virginica*	Nearctic Region	Chaloux, Andrea 2012
Noctuidae	*Pseudobryomima fallax* Hampson	*Pellaea andromedifolia*	Nearctic Region	Eiseman 2020b
Noctuidae	*Pseudobryomima muscosa* Hampson	*Polypodium californicum*	Nearctic Region	Eiseman 2020b
Tineidae	*Psychoides filicivora* Meyrick^12^	*Asplenium adiantum-nigrum*	Palaearctic Region	Gaedike 2019, Kim and Bae 2007
Tineidae	*Psychoides filicivora* Meyrick	*Asplenium ceterach*	Palaearctic Region	
Tineidae	*Psychoides filicivora* Meyrick	*Asplenium scolopendrium*	Palaearctic Region	
Tineidae	*Psychoides filicivora* Meyrick	*Asplenium trichomanes*	Palaearctic Region	
Tineidae	*Psychoides filicivora* Meyrick	*Dryopteris filix-mas*	Palaearctic Region	
Tineidae	*Psychoides filicivora* Meyrick	*Dryopteris aculeata*	Palaearctic Region	
Tineidae	*Psychoides filicivora* Meyrick	*Polystichum setiferum*	Palaearctic Region	
Tineidae	*Psychoides gosari* Kim & Bae	*Athyrium yokoscense*	Oriental and Palaearctic Regions	Kim and Bae 2007
Tineidae	*Psychoides gosari* Kim & Bae	*Dryopteris setosa*	Oriental and Palaearctic Region	
Tineidae	*Psychoides gosari* Kim & Bae	*Dryopteris chinensis*	Oriental and Palaearctic Regions	
Tineidae	*Psychoides gosari* Kim & Bae	*Dryopteris crassirhizoma*	Oriental and Palaearctic Regions	
Tineidae	*Psychoides gosari* Kim & Bae	*Dryopteris saxifraga*	Oriental and Palaearctic Regions	
Tineidae	*Psychoides phaedrospora* Meyrick^13^	Aspleniaceae	Palaearctic and Oriental Regions	Gaedike 2019, Kim and Bae 2007
Tineidae	*Psychoides verhuella* Bruand^14^	*Asplenium ceterach*	Palaearctic Region	Ellis 2020, Gaedike 2019, Heckford 2004, Kim and Bae 2007, Muus 2015
Tineidae	*Psychoides verhuella* Bruand	*Asplenium ruta-muraria*	Palaearctic Region	
Tineidae	*Psychoides verhuella* Bruand	*Asplenium scolopendrium*	Palaearctic Region	
Tineidae	*Psychoides verhuella* Bruand	*Asplenium trichomanes*	Palaearctic Region	
Tineidae	*Psychoides verhuella* Bruand	*Pteridium aquilinum*	Palaearctic Region	
Tortricidae	*Apoctena taipana* Felder & Rogenhofer	*Pyrrosia eleagnifolia*	Neozelandic Region	Patrick 2015
Tortricidae	*Celypha tiedemanniana* Zeller^15^	* Equisetum *	Palaearctic Region	Ellis 2020
Tortricidae	*Philocryptica polypodii* Watt	*Pyrrosia eleagnifolia*	Neozelandic Region	Patrick 2015
Cosmopterigidae	Hyposmocoma (Euperissus) ekaha Swezey	*Asplenium nidus*	Oriental Region	Kawahara et al. 2011
Cosmopterigidae	Hyposmocoma (Euperissus) trivitella Swezey	*Elaphoglossum aemulum*	Oriental Region	Kawahara et al. 2011
Cosmopterigidae	Hyposmocoma (Euperissus) trivitella Swezey	*Elaphoglossum gorgoneum*	Oriental Region	
Cosmopterigidae	Hyposmocoma (Euperissus) trivitella Swezey	*Elaphoglossum crassifolium*	Oriental Region	
Cosmopterigidae	Hyposmocoma (Euperissus) trivitella Swezey	*Elaphoglossum reticulatum*	Oriental Region	
Gelechiidae	*Monochroa harrisonella* Busck	*Pteridium aquilinum*	Nearctic Region	Eiseman 2020b
Gelechiidae	*Paltodora cytisella* Curti	*Pteridium aquilinum*	Palaearctic Region	Lawton 1976, Rigby and Lawton 1981
Hepialidae	*Endoclita excrescens* Butler*	*Equisetum arvense*	Palaearctic Region	Correia et al. 2020, Grehan 1989
Hepialidae	*Triodia sylvina* Linnaeus*	*Equisetum arvense*	Palaearctic Region	Correia et al. 2020, Grehan 1989
Psychidae	*Apterona helicoidella* Vallot	* Polypodium *	unknown	Alders and Gielis 1999
** Hymenoptera **				
Blasticotomidae	*Blasticotoma atra* Zhelochovtsev	unknown	unknown	Taeger et al. 2010, Wikipedia 2019
Blasticotomidae	*Blasticotoma filiceti* Klug	*Pteridium aquilinum*	Palaearctic Region	Ellis 2020, Liston 2007, Novgorodova and Biryukova 2011, Shcherbakov 2006, Shcherbakov 2008, Taeger et al. 2010, Wikipedia 2019
Blasticotomidae	*Blasticotoma filiceti* Klug	* Dryopteris *	Palaearctic Region	
Blasticotomidae	*Blasticotoma filiceti* Klug	* Polystichum *	Palaearctic Region	
Blasticotomidae	*Blasticotoma filiceti* Klug	*Matteuccia struthiopteris*	Palaearctic Region	
Blasticotomidae	*Blasticotoma filiceti* Klug	*Athyrium alpestre*	Palaearctic Region	
Blasticotomidae	*Blasticotoma filiceti* Klug	*Athyrium filix-femina*	Palaearctic Region	
Blasticotomidae	Blasticotoma filiceti var. pacificus Malaise	unknown	unknown	Taeger et al. 2010, Wikipedia 2019
Blasticotomidae	*Blasticotoma nipponica* Takeuchi	unknown	unknown	Wikipedia 2019, Taeger et al. 2010
Blasticotomidae	*Blasticotoma smithi* Shinohara	unknown	unknown	Taeger et al. 2010, Wikipedia 2019
Blasticotomidae	*Blasticotoma warabii* Togashi	unknown	unknown	Taeger et al. 2010, Wikipedia 2019
Blasticotomidae	*Bohea abrupta* Maa	unknown	unknown	Taeger et al. 2010, Wikipedia 2019
Blasticotomidae	*Paremphytus ostentus* Brues	unknown	unknown	Taeger et al. 2010, Wikipedia 2019
Blasticotomidae	*Runaria flavipes* Takeuchi	unknown	unknown	Taeger et al. 2010, Wikipedia 2019
Blasticotomidae	*Runaria hunannica* Wei in Wei & Nie	unknown	unknown	Taeger et al. 2010, Wikipedia 2019
Blasticotomidae	*Runaria punctata* Wei in Wei & Nie	unknown	unknown	Taeger et al. 2010, Wikipedia 2019
Blasticotomidae	*Runaria shaanxinica* Wei in Wei & Nie	unknown	unknown	Taeger et al. 2010, Wikipedia 2019
Blasticotomidae	*Runaria taiwana* Shinohara	unknown	unknown	Taeger et al. 2010, Wikipedia 2019
Tenthredinidae	*Aneugmenus coronatus* Klug	*Pteridium aquilinum*	Palaearctic Region	Beneš 2014, Ellis 2020, Schwarz 2005
Tenthredinidae	*Aneugmenus coronatus* Klug	*Dryopteris filix-mas*	Palaearctic Region	
Tenthredinidae	*Aneugmenus coronatus* Klug	*Polystichum setiferum*	Palaearctic Region	
Tenthredinidae	*Aneugmenus coronatus* Klug	*Athyrium filix-femina*	Palaearctic Region	
Tenthredinidae	*Heptamelus dahlbomi* Thomson	*Athyrium filix-femina*	Nearctic and Palaearctic Regions	Vikberg and Liston 2009
Tenthredinidae	*Heptamelus ochroleucus* Stephens^16^	*Blechnum spicant*	Nearctic and Palaearctic Regions	Ellis 2020, Shcherbakov 2008, Vikberg 2017, Vikberg and Liston 2009
Tenthredinidae	*Heptamelus ochroleucus* Stephens	*Matteuccia struthiopteris*	Nearctic and Palaearctic Regions	
Tenthredinidae	*Heptamelus ochroleucus* Stephens	*Dryopteris dilatata*	Nearctic and Palaearctic Regions	
Tenthredinidae	*Heptamelus ochroleucus* Stephens	*Polypodium vulgare*	Nearctic and Palaearctic Regions	
Tenthredinidae	*Heptamelus ochroleucus* Stephens	*Athyrium filix-femina*	Nearctic and Palaearctic Regions	
** Coleoptera **				
Buprestidae	*Endelus bakerianus* Obenberger	*Lygodium microphyllum*	Oriental Region	Kalashian 2013, Goolsby et al. 2003, Mehltreter et al. 2010
Buprestidae	*Neotrachys bellamyi* Hespenheide	*Gleichenia glauca*	Neotropical Region	Hespenheide 2006
Buprestidae	*Neotrachys bicolor* Hespenheide	*Cnemidaria petiolata*	Neotropical Region	Hespenheide 1982
Buprestidae	*Neotrachys bordoni* Cobos	Cyatheaceae	Neotropical Region	Hespenheide 1982
Buprestidae	*Neotrachys caerulea* Hespenheide	Cyatheaceae	Neotropical Region	Hespenheide 1982
Buprestidae	*Neotrachys concinna* Fisher	Cyatheaceae	Neotropical Region	Hespenheide 1982, Hespenheide 2006
Buprestidae	*Neotrachys cyanipennis* Fisher	Cyatheaceae	Neotropical Region	Hespenheide 2006
Buprestidae	*Neotrachys estebana* Kerremans*	* Dicranopteris *	Neotropical Region	Hespenheide 1982
Buprestidae	*Neotrachys fennahi* Thery	Cyatheaceae	Neotropical Region	Hespenheide 1980
Buprestidae	*Neotrachys gleicheniae* Hespenheide	* Gleichenia *	Neotropical Region	Hespenheide 1982
Buprestidae	*Neotrachys hoffmani* Fisher	Cyatheaceae	Neotropical Region	Hespenheide 1980, Hespenheide 1982
Buprestidae	*Neotrachys mariae* Hespenheide	* Gleichenia *	Neotropical Region	Hespenheide 2006
Buprestidae	*Neotrachys resplendens* Hespenheide	Cyatheaceae	Neotropical Region	Hespenheide 1982
Buprestidae	*Neotrachys segregata* Waterhouse	Gleicheniaceae*	Neotropical Region	Hespenheide 1982
Buprestidae	*Neotrachys solisi* Hespenheide	* Gleichenia *	Neotropical Region	Hespenheide 2006
Chrysomelidae	*Febra insularis* Bryant	*Acrostichum aureum*	Oriental Region	Samuelson 1973, Santiago-Blay 2004
Chrysomelidae	*Febra ovata* Bryant	*Angiopteris evecta*	Oriental Region	Samuelson 1973, Nadein 2013, Jolivet 1991
Chrysomelidae	*Febra venusta* Clark	* Nephrolepis *	Oriental Region	Samuelson 1973, Santiago-Blay 2004
Chrysomelidae	*Halticorcus bhaumiki* Basu et Sengupta^17^	*Pteris vittata*	Palaearctic and Oriental Regions	Isowa and Kojima 2011, Konstantinov and Prathapan 2008, Patra and Bera 2007
Chrysomelidae	*Halticorcus bhaumiki* Basu et Sengupta	*Ampelopteris prolifera*	Palaearctic and Oriental Regions	
Chrysomelidae	*Halticorcus bhaumiki* Basu et Sengupta	* Cyclosorus *	Palaearctic and Oriental Regions	
Chrysomelidae	*Halticorcus bhaumiki* Basu et Sengupta	*Christella dentata*	Palaearctic and Oriental Regions	
Chrysomelidae	*Halticorcus bhaumiki* Basu et Sengupta	*Nephrolepis cordifolia*	Palaearctic and Oriental Regions	
Chrysomelidae	*Halticorcus bhaumiki* Basu et Sengupta	*Nephrolepis exaltata*	Palaearctic and Oriental Regions	
Chrysomelidae	*Halticorcus bhaumiki* Basu et Sengupta	*Adiantum lunulatum*	Palaearctic and Oriental Regions	
Chrysomelidae	*Halticorcus bhaumiki* Basu et Sengupta	*Drynaria propinqua*	Palaearctic and Oriental Regions	
Chrysomelidae	*Halticorcus bhaumiki* Basu et Sengupta	*Pyrrosia adnascens*	Palaearctic and Oriental Regions	
Chrysomelidae	*Halticorcus bhaumiki* Basu et Sengupta	*Microsorum scolopendria*	Palaearctic and Oriental Regions	
Chrysomelidae	*Halticorcus hiranoi* Takizawa^18^	*Lemmaphyllum microphyllum*	Palaearctic Region	Kato 1991, Santiago-Blay 2004
Chrysomelidae	*Halticorcus hiranoi* Takizawa	Loxogramme salicifolia*	Palaearctic Region	
Chrysomelidae	*Halticorcus kasuga* Nakane	*Lepisorus thunbergianus*	Palaearctic Region	Isowa and Kojima 2011
Chrysomelidae	*Halticorcus kasuga* Nakane	*Lepisorus onoei*	Palaearctic Region	
Chrysomelidae	*Halticorcus kasuga* Nakane	*Lemmaphyllum microphyllum*	Palaearctic Region	
Chrysomelidae	*Halticorcus kasuga* Nakane	*Pyrrosia linearifolia*	Palaearctic Region	
Chrysomelidae	*Halticorcus platycerii* Lea	*Platycerium alcicorne*	Australotropical, Australotemperate and Palaearctic Regions	Hawkeswood 2003, Isowa and Kojima 2011, Sinclair and Hughes 2010
Chrysomelidae	*Halticorcus platycerii* Lea	*Asplenium nidus*	Australotropical, Australotemperate and Palaearctic Regions	
Chrysomelidae	*Halticorcus sauteri* Chen^19^	*Colysis elliptica*	Palaearctic Region	Kato 1991, Santiago-Blay 2004
Chrysomelidae	*Halticorcus sauteri* Chen	*Leptochilus ellipticus*	Palaearctic Region	
Chrysomelidae	*Hippuriphila babai* Chujo*	* Equisetum *	Palaearctic Region	Correia et al. 2020, Poinar 2014
Chrysomelidae	*Hippuriphila canadensis* Brown*	*Equisetum arvense*	Nearctic Region	Correia et al. 2020, Poinar 2014
Chrysomelidae	*Hippuriphila catherinae* Barr*	* Equisetum *	Neotropical Region	Correia et al. 2020, Poinar 2014
Chrysomelidae	*Hippuriphila equiseti* Beller et Hatch*	*Equisetum arvense*	Nearctic Region	Correia et al. 2020, Poinar 2014
Chrysomelidae	*Hippuriphila modeeri* Linnaeus	*Equisetum arvense*	Palaearctic Region	Biological Records Centre 2020, Ellis 2020, Santiago-Blay 2004
Chrysomelidae	*Hippuriphila modeeri* Linnaeus	*Equisetum fluviatile*	Palaearctic Region	
Chrysomelidae	*Hippuriphila modeeri* Linnaeus	*Equisetum palustre*	Palaearctic Region	
Curculionidae	*Bagous claudicans* Boheman	*Equisetum fluviatile*	except for Central and South America, all the world	Ellis 2020, Gosik et al. 2019
Curculionidae	*Bagous lutulentus* Gyllenhal^20^	*Equisetum fluviatile*	except for Central and South America, all the world	Ellis 2020, Gosik 2009, Gosik et al. 2019, Gosik and Wanat 2014
Curculionidae	*Grypus brunnirostris* Fabricius^21^	*Equisetum arvense*	Nearctic Region	Ellis 2020, George 2014
Curculionidae	*Grypus brunnirostris* Fabricius	*Equisetum fluviatile*	Nearctic Region	
Curculionidae	*Grypus brunnirostris* Fabricius	*Equisetum ramosissimum*	Nearctic Region	
Curculionidae	*Grypus equiseti* Fabricius^22^	*Equisetum arvense*	Nearctic and Palaearctic Regions	Ellis 2020, George 2014, Gosik et al. 2019
Curculionidae	*Grypus equiseti* Fabricius	*Equisetum palustre*	Nearctic and Palaearctic Regions	
Curculionidae	*Grypus equiseti* Fabricius	*Equisetum pratense*	Nearctic and Palaearctic Regions	
Curculionidae	*Grypus equiseti* Fabricius	*Equisetum sylvaticum*	Nearctic and Palaearctic Regions	
Curculionidae	*Stenopelmus rufinasus* Gyllenhal	* Azolla *	Nearctic, Afrotopical and Palaearctic Regions	Center et al. 2002, Hill and Cilliers 1999, Richerson and Grigarick 1967

* possible host fern or miner

Synonyms:

1 *Chromatomyia cheilanthus* Garg = *Phytomyza cheilanthus* Garg;

2 *Chromatomyia dorsata* Hendel = *Phytomyza dorsata* Hendel;

3 *Chromatomyia dryoptericola* Sasakawa = *Phytomyza dryoptericola* Sasakawa

4 *Chromatomyia scolopendri* Robineau-Desvoidy = *Phytomyzascolopendri Goureau* = *Phytomyza elegans* Goureau = *Phytomyza nevadensis* Strobl = *Chromatoinyia nevadensis* Strobl;

5 *Liriomyza equiseti* de Meijere = *Liriomyza kenti* Spencer;

6 *Liriomyza occipitalis* Hendel = *Liriomyza bruscae* Hering;

7 *Liriomyza virgo* Zetterstedt = *Liriomyza arcticola* Spencer = *Phytomyza pallicornis* Zetterstedt;

8 *Liriomyza virgula* Frey = *Liriomyza larissa* Hering;

9 *Chirosia cinerosa* Zetterstedt = *Pycnoglossa cinerosa* Zetterstedt;

10 *Chirosia grossicauda* Strobl = *Chirosia parvicornis* nec Zetterstedt;

11 *Chirosia histricina* Rondani = *Chirosia setifemur* Ringdahl = *Pycnoglossa hystrix* Brischke = *Pycnoglossa hystricina*;

12 *Psychoides filicivora* Meyrick = *Teichobia filicivora* Meyrick;

13 *Psychoides phaedrospora* Meyrick = *Mnesipatris phadrospora* Meyrick;

14 *Psychoides verhuella* Bruand = *Teichobia verhuellella* Herrich-Schaffer = *Lambrosetia verhuellella* Stainton;

15 *Celypha tiedemanniana* Zeller = *Olethreutes tiedemanniana* Zeller Kuznetsov = *Syricoris tiedemanniana* Zeller;

16 *Heptamelus ochroleucus* Stephens = *Melicerta ochroleucus* Stephens;

17 *Halticorcus bhaumiki* Basu et Sengupta = *Schenklingia bhaumiki* Basu and Sengupta;

18 *Halticorcus hiranoi* Takizawa = *Schenklingia hiranoi* Takizawa;

19 *Halticorcus sauteri* Chen = *Schenklingia sauteri* Chen;

20 *Bagous lutulentus* Gyllenhal = *Bagous glabrirostris var. nigritarsis* Thomson;

21 *Grypus brunnirostris* Fabricius = *Curculio Inspectionatus* Herbst;

22 *Grypus equiseti* Fabricius = *Grypidius equiseti* Fabricius;
